# A New Method for Quantitative Immunoblotting of Endogenous α-Synuclein

**DOI:** 10.1371/journal.pone.0081314

**Published:** 2013-11-20

**Authors:** Andrew J. Newman, Dennis Selkoe, Ulf Dettmer

**Affiliations:** From the Center for Neurologic Diseases, Brigham and Women’s Hospital and Harvard Medical School, Boston, Massachusetts, United States of America; Hertie Institute for Clinical Brain Research and German Center for Neurodegenerative Diseases, Germany

## Abstract

β-Sheet-rich aggregates of α-synuclein (αSyn) are the hallmark neuropathology of Parkinson’s disease and related synucleinopathies, whereas the principal native structure of αSyn in healthy cells - unfolded monomer or α-helically folded oligomer - is under debate. Our recent crosslinking analysis of αSyn in intact cells showed that a large portion of endogenous αSyn can be trapped as oligomers, most notably as apparent tetramers. One challenge in such studies is accurately quantifying αSyn Western blot signals among samples, as crosslinked αSyn trends toward increased immunoreactivity. Here, we analyzed this phenomenon in detail and found that treatment with the reducible amine-reactive crosslinker DSP strongly increased αSyn immunoreactivity even after cleavage with the reducing agent β-mercaptoethanol. The effect was observed with all αSyn antibodies tested and in all sample types from human brain homogenates to untransfected neuroblastoma cells, permitting easy detection of endogenous αSyn in the latter, which had long been considered impossible. Coomassie staining of blots before and after several hours of washing revealed complete retention of αSyn after DSP/β-mercaptoethanol treatment, in contrast to a marked loss of αSyn without this treatment. The treatment also enhanced immunodetection of the homologs β- and γ-synuclein and of histones, another group of small, lysine-rich proteins. We conclude that by neutralizing positive charges and increasing protein hydrophobicity, amine crosslinker treatment promotes adhesion of αSyn to blotting membranes. These data help explain the recent report of fixing αSyn blots with paraformaldehyde after transfer, which we find produces similar but weaker effects. DSP/β-mercaptoethanol treatment of Western blots should be particularly useful to quantify low-abundance αSyn forms such as extracellular and post-translationally modified αSyn and splice variants.

## Introduction

The synuclein family consists of α-, β- and γ-Synuclein (αSyn, βSyn, γSyn), small soluble proteins with a molecular weight below 15 kDa. αSyn is by far the best known and most studied among the three, due to its strong link with Parkinson’s disease (PD), the second most common human neurodegenerative disease. Point mutations and copy number variants in the αSyn gene cause familial forms of PD [1]–[4], and αSyn is the major component of Lewy bodies, intraneuronal protein aggregates that are the cytopathological hallmark of both familial and sporadic PD [5]. Its central role in PD pathogenesis has attracted considerable research into the biochemical, biophysical and functional properties of αSyn. However, despite more than two decades of αSyn research, essential features, such as molecular function, interaction partners, structure, and even sub-cellular localization, are still contentious. Recent developments indicate that results of studies of αSyn are dependent on the model system used: standard recombinant expression of αSyn in bacteria, for example, provides large quantities of protein suitable for biophysical and structural analyses, but this protein may insufficiently replicate the posttranslational modifications, folding and oligomerization of the endogenous protein in neurons [6]–[10]. Analysis of αSyn in eukaryotic cells, on the other hand, is limited by relatively low abundance and various technical challenges. For example, Western blot analysis of endogenous αSyn in immortalized cell lines, even those of neuronal origin such as SH-SY5Y, had long been considered almost impossible due to extremely low expression levels [11]. A recent publication, however, has challenged this view by demonstrating that clear and strong detection of total endogenous αSyn from cultured cells is facilitated by fixation of proteins on blotted membranes with low concentrations of paraformaldehyde (PFA) [12], a method previously applied to the study of hemoglobin [13]. The authors suggest that this treatment prevents washing off of synuclein from PVDF or nitrocellulose membranes. We applied their method in a previous study of αSyn crosslinking, and it enabled meaningful Western blot comparisons of monomeric and oligomeric αSyn species without vs. with crosslinking [12]. Here, we report that immunodection of αSyn monomer can be improved even further – by more than 100% – with the application of reducible amine-reactive crosslinkers such as dithiobis[succinimidylpropionate] (DSP) followed by reductive cleavage (5% βME), prior to SDS-PAGE and electroblotting. In a variety of samples from neuroblastoma cell lines to human brain, we demonstrate that our novel method of improved αSyn monomer immunodetection is superior to the PFA treatment of blots. Purified recombinant αSyn transferred to PVDF membranes and stained with Coomassie dye before and after hours of washing was virtually 100% retained on the membrane with DSP/βME treatment, while untreated αSyn was nearly completely lost. Moreover, we observed the same enhancement of monomer immunodetection with the synuclein homologs β- and γSyn, as well as with another group of small, lysine-rich proteins, the histone family, suggesting that this new method may also benefit work outside the field of αSyn research.

## Materials and Methods

### Cell culture

All materials were purchased from Invitrogen unless stated otherwise. All cells were cultured at 37°C in a 5% CO_2_ atmosphere. Human erythroid leukemia cells (HEL; ATCC number TIB-180) were cultured in RPMI 1640 (ATCC modification) supplemented with 10% fetal bovine serum (Sigma), 10 units/mL penicillin and 10 µg/mL streptomycin (Pen-Strep). Human neuroblastoma cells BE(2)-M17 (called M17D; ATCC number CRL-2267) and 3D5, an M17D-derived line overexpressing wild type human αSyn regulated by the tetracycline-off system [14], were cultured in high-glucose DMEM supplemented with 10% FBS, 2 mM L-glutamine and Pen-Strep. Doxycycline was added to cell medium to a final concentration of 100 ng/mL to repress αSyn expression. The human neuroblastoma cell line SH-SY5Y (ATCC number CRL-2266) was cultured in a 1∶1 mixture of high-glucose DMEM and F-12 supplemented with 10% FBS, 2 mM L-glutamine and Pen-Strep.

### Sample preparation from cultured cells and brain tissues

Our method of generation of cytosolic lysates from cultured human cell lines and primary rat neurons has been described [7]. In this study, membranes were routinely pelleted by centrifugation at 100,000 *g* for 60 min at 4°C. Brain homogenates from frozen mouse brain and human brain were generated by thawing, dounce homogenization and 15 s sonication (Sonic Dismembrator model 300, Fisher Scientific, Waltham, MA; microtip setting  =  40). Brains were dissected from C57BL/6 male mice aged 11–13 weeks (Jackson Laboratory, Bar Harbor, ME) and stored at –80°C. A sample of human cerebral cortex from a neurologically normal subject was stored at –80°C.

### Ethics Statement

The use of human brain samples was approved under protocol number 1999P001180 ('Aging in the Brain: Role of the Fibrous Proteins') by the Partners Human Research Committee (PHRC), the Institutional Review Board (IRB) of Partners Research Management. Informed written consent for the use of post-mortem brain tissue was obtained from the families donating the patients' brains to research when they enrolled in our CND Tissue Donation Program. The neuropathologist obtained consent at the time of autopsy. Rodent samples were acquired under protocol number 05022 ('Mouse Models for Parkinson's Disease'), approved by the appropriate IACUC, the Harvard Medical Area Standing Committee on Animals.

### Crosslinkers

Dithiobis[propionimidate] (DTBP), dithiobis[succinimidylpropionate] (DSP) and disuccinimidylglutarate (DSG) were from Pierce. Crosslinkers were dissolved in DMSO to a 50X stock concentration prior to addition to sample. Detailed crosslinking protocols (*in vivo* and *in vitro* with cells and on recombinant protein) are in [7].

### Electrophoresis & Immunoblotting

All materials were purchased from Invitrogen unless stated otherwise. Total protein concentrations were determined by BCA assay (Thermo Scientific) according to manufacturer’s directions. Samples were prepared for electrophoresis by diluting with 4X NuPAGE sample buffer with or without 20% β-mercaptoethanol followed by boiling for 10 min. Routinely, 15–30 µg of sample were electrophoresed on NuPAGE 4–12% Bis-Tris gels with NuPAGE MES-SDS running buffer and the SeeBlue Plus2 molecular weight markers. After electrophoresis, gels were blotted onto Immobilon-PSQ 0.2-µm PVDF (Millipore) for 90 min at 400 mA constant current at 4°C in 25 mM Tris, 192 mM glycine, 20% methanol transfer buffer. Unless stated otherwise, post-transfer membranes were treated with 0.4% paraformaldehyde (PFA) in PBS for 30 min at RT and then rinsed with water [12]. Blocking was in 0.2% iBlock (Applied Biosystems) in PBS with 0.1% (v/v) Tween-20 (PBST) for ≥30 min. Membranes were then incubated in primary antibody in 0.2% iBlock in PBST with 0.02% sodium azide for either 1 h at RT or overnight at 4°C. Membranes were then washed 3×10 min in PBST and incubated in secondary antibody in 0.2% iBlock in PBST followed by washing 3×10 min in PBST and developing with ECL Prime (GE Healthcare-Amersham Biosciences) according to the manufacturer’s instructions. To visualize proteins on PVDF, membranes were briefly incubated in Ponceau solution or Coomassie (PageBlue Protein Staining Solution, Thermo Scientific; application on dried membranes).

### Antibodies

2F12, a monoclonal antibody (mAb) to αSyn, was produced in-house [7]. Other αSyn mAbs were 15G7 [15] and Syn1 (Clone 42, Becton-Dickinson), and polyclonal antibody (pAb) C20 (Santa Cruz) was also used. Other antibodies were mAb EP1537Y to β-synuclein (Novus Biologicals), pAb 6169 to γ-synuclein (Abcam), mAb 8226 to β-actin (Abcam), mAb AA2 to β-tubulin (Sigma), mAb to calmodulin (05-173, Millipore), mAb 71.1 to GAPDH (Sigma), pAb to DJ-1 ([16]), pAb to RAN (4462, Cell Signaling), pAb to 14-3-3 (9063, Abcam), pAb to UCH-L1 (ab1761, Millipore), pAb to Histone H3 (9715, Cell Signaling), and pAb to trimethyl Histone H3 Lys27 (ABE44, EMD Millipore).

### Semiquantitave densitometry of Western blot signals

Scanned Western blots were analyzed with ImageJ software, version 1.47 [17]. Pictures were inverted and background signal from an empty lane subtracted to obtain the actual signals for each lane.

## Results

### Discrepancy between immunoblotting of αSyn from crosslinker-treated and untreated lysates

**Figure 1:**
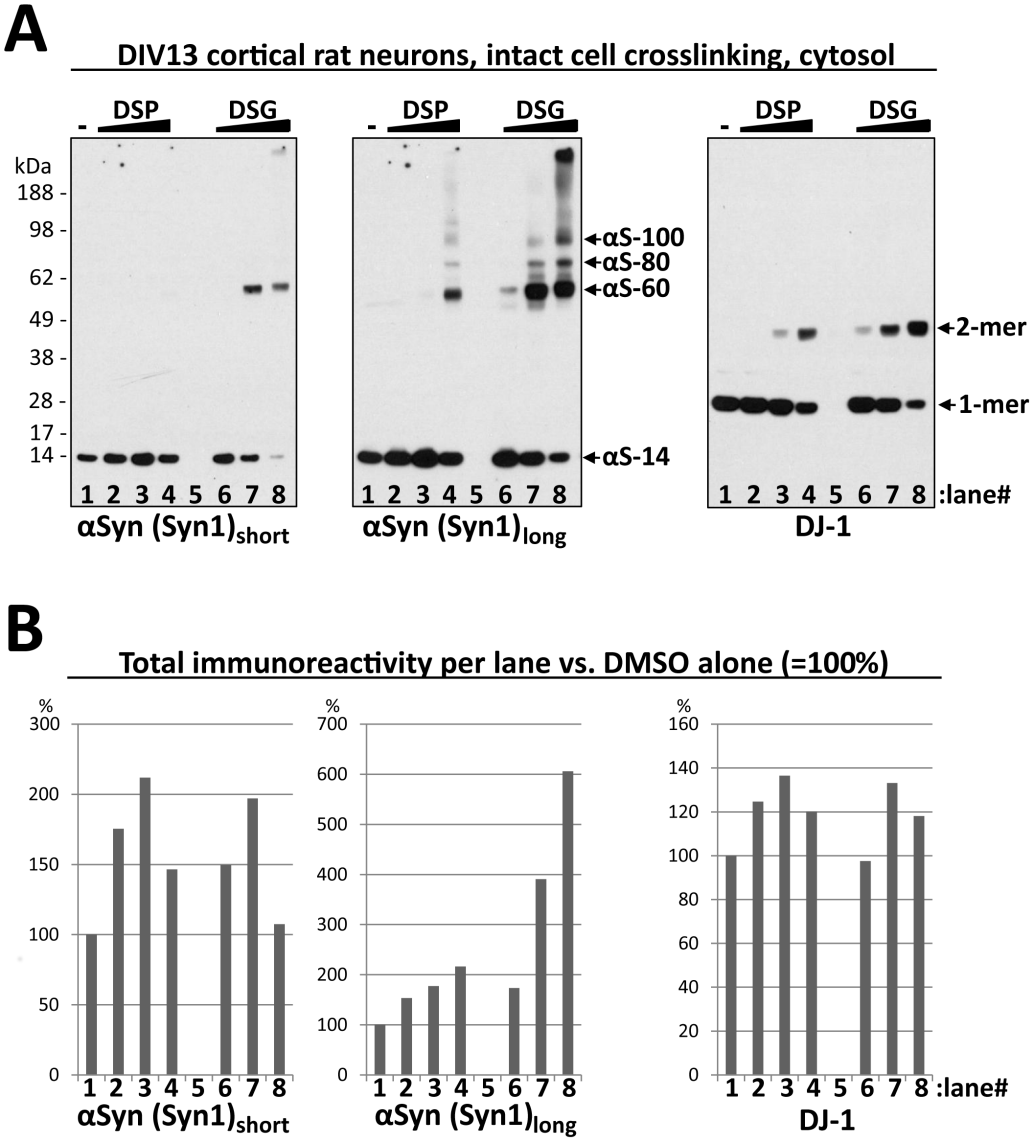
Discrepancy between immunoblotting of αSyn from crosslinker-treated and untreated lysates. **A.** Intact primary cortical rat neurons (13 days *in vitro*) were incubated with DMSO only (-) or a gradient of 0.1, 0.5 and 1.0 mM DSP or DSG, sonicated in PBS supplemented with protease inhibitors and spun at 100,000 *g* for 60 min. The resultant cytosols (30 µg) were blotted with αSyn antibody Syn1 (left panel: short exposure; middle panel: long exposure) and with anti-DJ-1 as a control (right panel). Arrows indicate the crosslinker-trapped αSyn species that have been described before [7] as well as the DJ-1 dimer (2-mer) and monomer (1-mer). **B.** Densitometric analysis of the blots shown in Fig. 1a using ImageJ (see Materials and Methods). Bars indicate the total immunoreactivity of each lane as a percentage of the signal from DMSO-only treated sample (lane 1), after subtraction of the background from the empty lane 5.

We recently published a detailed crosslinking analysis of αSyn in intact cells [7] that led us to conclude that a large portion of endogenous cytosolic αSyn exists in physiological oligomers, principally apparent tetramers, consistent with previous studies from our lab [6] and others [8]–[10]. In contrast to these findings, some labs have recently published evidence that leads them to support the earlier model that physiological αSyn exists in cells and tissues principally as unfolded monomers. However, these studies either did not include the intact-cell crosslinking approach [18], or the trapping of αSyn at oligomeric (e.g., dimeric) positions by crosslinking was considered unspecific [19]. One challenge in studying αSyn by crosslinking is achieving equality of Western blot signals between crosslinker- and control-treated samples. In most of our experiments, the crosslinker-modified αSyn showed somewhat greater immunoreactivity than did non-crosslinked αSyn [7]. We speculated that unmodified αSyn may be washed off of Western blot membranes, whereas crosslinker modification might prevent this loss. This hypothesis emerged from the finding of Lee and Kamitani that 0.4% paraformaldehyde (PFA) fixing of blot membranes prevented the apparent loss of αSyn monomers during immunoblotting, thereby enhancing their immunodetection [12]. By including their PFA step in our standard protocol for αSyn crosslinking analysis, we achieved quantitatively meaningful results (‘conservation of αSyn immunochemical matter’) to a much greater extent: the combined immunoreactivity of all αSyn species after crosslinking roughly approximated that of the monomer alone before crosslinking. However, even with the routine use of PFA treatment, we still found some inconsistencies in the relative αSyn immunoreactivities of untreated and crosslinker-treated samples. For example, we observed certain trends in αSyn immunodetection, as exemplified by primary rat neurons (Fig. 1a, two left panels are short and long exposures). First, treatment of intact neurons with increasing amounts of the reducible amine crosslinker DSP resulted in a stepwise increase in αSyn monomer immunoreactivity (lanes 2 and 3) compared to the vehicle (DMSO) treated control (lane 1), until at a higher DSP concentration, αS-60 (αSyn tetramer) was trapped (lane 4). Second, low amounts of the non-reducible amine crosslinker DSG trapped small amounts of αSyn oligomers but still increased monomer detection (lane 6) compared to DMSO alone (lane 1). Third, a higher DSG concentration trapped more oligomeric αSyn (lane 7), but this increase was not accompanied by a corresponding decrease in detected monomer compared to DMSO alone (lane 1). Fourth, an even higher DSG concentration led to appearance of a high MW αSyn-reactive smear, primarily at the expense of the monomer signal (lane 8). Densitometry of these blots (Fig. 1b, left and middle panel) supported these qualitative impressions and suggested that the relative immunoreactivity of the crosslinker-treated and untreated samples that one observes depends on the exposure time of the blot. Most importantly, longer blot exposures reveal the presence of HMW smears at higher DSG concentrations, thus exaggerating the total αSyn immunoreactivity in those lanes (see lane 8). A parallel analysis for the well-known dimeric protein DJ-1 revealed better conservation of total immunoreactivity between the treatments for this control protein and no appearance of high MW smears (Western blot: Fig. 1a, right panel; densitometry: Fig. 1b, right panel).

### Lysine modification increases αSyn immunoreactivity by strengthening attachment to blot membranes

**Figure 2:**
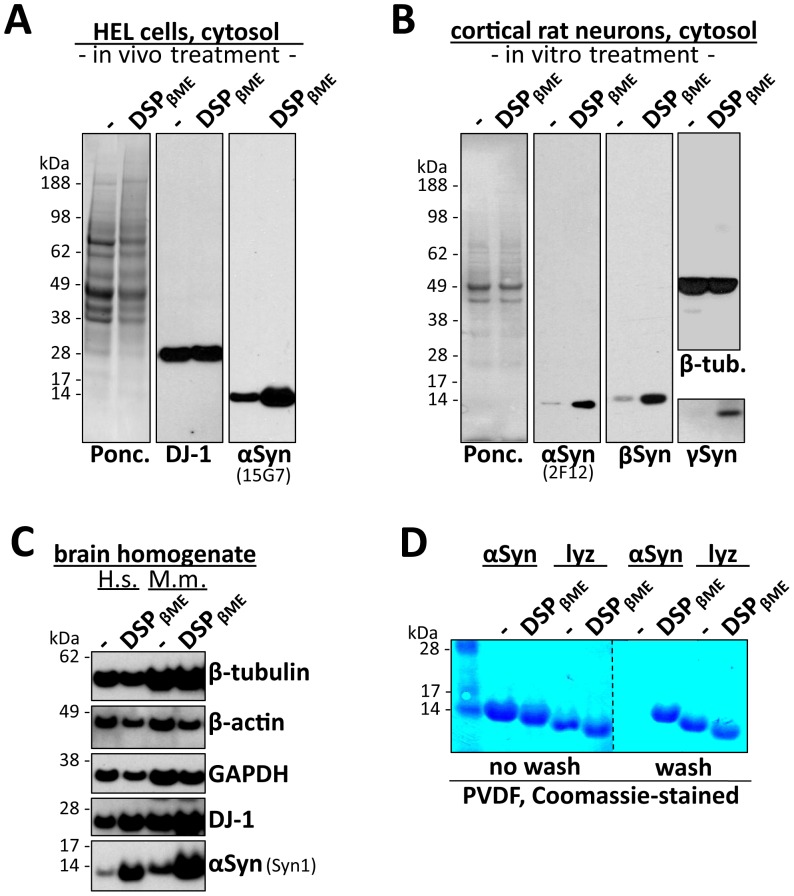
Lysine modification increases αSyn immunoreactivity by strengthening attachment to blot membranes. **A.** HEL cells were crosslinked *in vivo* with the cleavable crosslinker DSP (1 mM) in comparison to DMSO-only treatment (-). Cytosols were prepared (post-20,000 *g*) and boiled in sample buffer with 5% (v/v) β-mercaptoethanol (βME), which cleaves the disulfide bond of DSP (DSP_ßME_: reduced DSP). Samples (30 µg) were blotted with αSyn antibody 15G7. Equal protein loading was visualized by blotting for DJ-1 and by Ponceau (Ponc.) staining. **B.** Primary cortical rat neurons (13 days *in vitro*) were treated with 1 mM DSP *in vitro* (cytosolic lysates) in comparison to DMSO-only (-) treatment. Cytosols (post 100,000 *g*) were boiled in sample buffer with 5% βME and blotted with αSyn antibody 2F12, as well as antibodies to βSyn, γSyn and β-tubulin (β-tub.). Ponceau (Ponc.) stain of the blot membrane is shown on the left. **C.** Human (H.s.; 15 µg) and mouse (M.m., 30 µg) brain homogenates (PBS soluble fraction; post 100,000 *g*) were treated *in vitro* with DSP or DMSO-only (-), followed by boiling in sample buffer containing 5% βME. Blots were developed with αSyn antibody Syn1 as well as antibodies to β-tubulin, β-actin, GAPDH and DJ-1. **D.** Purified recombinant αSyn and purified hen-egg lysozyme (lyz) were crosslinked at a concentration of 500 ng/µL with 2 mM DSP (DSP_ßME_) in comparison to DMSO only (-), followed by boiling in sample buffer with 5% βME. After separation by SDS-PAGE, samples were transferred to PVDF membranes which were dried immediately (left panel: ‘no wash’) or after washing overnight in PBST (‘wash’). Dried membranes were briefly stained with Coomassie Blue. 14, 17 and 28 kDa molecular weight markers are visible in the outer left lane.

As Western blotting is only a semi-quantitative method, given the potential for signal saturation and overestimation of diffuse immunoreactivity, the increase in total αSyn immunoreactivity in the crosslinked samples was not unexpected, particularly in those with larger amounts of trapped oligomers and upon long exposure times. Nevertheless, the increase in αSyn monomer signal at crosslinker amounts that trapped only trace amounts of αSyn oligomers (Fig. 1a, lanes 2, 3 and 6) was unanticipated. This phenomenon was reminiscent of our difficulty in achieving equality of Western blot signals between crosslinker- and control-treated samples when initially blotting for αSyn without PFA treatment. We therefore reasoned that Western blotting preferentially detects crosslinker-modified αSyn, whether monomeric or oligomeric, even when PFA fixation is used. We hypothesized that PFA treatment of blots alone does not fully prevent αSyn from washing off during development, whereas robust crosslinker-modification of the lysine residues in the protein may be sufficient. To test this, we treated human erythroid leukemia (HEL) cells (which express high endogenous levels of αSyn [7]) with 1 mM of DSP *in vivo* or DMSO only, then generated high-speed cytosols, normalized for protein concentration, and cleaved the reducible crosslinker by adding 5% βME before samples were boiled for SDS-PAGE. By Western blot analysis on PFA treated blots, we observed a markedly enhanced signal for αSyn in the DSP/βME-treated sample (Fig. 2a, right panel). Importantly the control protein DJ-1 did not show a similar effect (Fig. 2a, middle panel), and Ponceau staining of the PVDF membrane did not indicate higher protein loading in the DSP/βME lane (left panel). We then tested our paradigm on the primary neurons and found that in addition to *in vivo* (not shown) even *in vitro* DSP treatment (addition of DSP to cytosolic lysates) followed by βME cleavage enhanced the αSyn immunosignal (Fig. 2b, second panel from the left, compare left and right lanes). Signal for the control monomeric protein β-tubulin (Fig. 2b, right panel, upper part) was not affected by DSP treatment, but βSyn and γSyn (Fig. 2b, third panel from the left and right panel, bottom part) showed similar marked enhancement of monomer by DSP/βME as did αSyn. Ponceau staining (Fig. 2b, left panel) and blotting for β-tubulin (Fig. 2b, right panel, top part) ruled out higher protein loading in the DSP/βME lanes, as expected.

Our next step was to test the DSP/βME treatment on the αSyn protein source most relevant to the study of Parkinson’s disease, human brain (Fig. 2c, left two lanes labled ‘H.s.’). Again, we observed a strong signal enhancement for αSyn monomers with DSP/βME treatment of brain homogenates (Fig. 2c, lowest panel). A similar effect was achieved with mouse brain homogenates (Fig. 2c, right two lanes labled ‘M.m.’). The control proteins β-tubulin and β-actin behaved differently, as their signals were unaffected (β-tubulin) or decreased (β-actin) by DSP/βME treatment. Based on the PFA result of Lee and Kamitani [12], we had initially hypothesized that increased immunodetection of αSyn by improved blotting procedures was a result of the αSyn protein not washing off the blot. However, alternate explanations such as enhanced antibody accessibility to αSyn were possible although unlikely, as all αSyn antibodies tested so far led to similar results. To rule out this possibility by avoiding the need for immunodetection, we turned to pure αSyn protein. Purified recombinant αSyn [20] and purified hen-egg lysozyme as a control [7] were incubated with DSP/βME or DMSO alone (Fig. 2d). All four conditions (two proteins, each with two treatments) were subjected to SDS-PAGE followed by transfer to a PVDF membrane. One part of the membrane (containing all four samples) was dried immediately after transfer, reducing the risk of subsequent protein wash-off (Fig. 2d, left panel). The other half of the membrane (also containing all 4 samples) was incubated overnight in our standard wash buffer (PBS with 0.1% Tween-20; PBST), then briefly washed in water and dried as well (Fig. 2d, right panel). We visualized the proteins on the dried membranes by brief incubation in Coomassie (see Materials and Methods), which we identified as the best method because it stained untreated and DSP/βME-treated αSyn similarly well. (Ponceau staining underestimated protein in crosslinker-treated samples, as it binds to positive charges, which are neutralized by the crosslinker; see Fig. 2a.). We found that unmodified αSyn, while fully present immediately after transfer (Fig. 2d, left half), was almost entirely gone after overnight washing of the blot (right half). In striking contrast, DSP/βME-treated αSyn was detectable to a similar extent with and without overnight washing. The control protein, lysozyme, was largely unaffected by any of the conditions. We conclude that the enhancing effect of DSP/βME on αSyn immunodetection is indeed a result of less washing-off of the crosslinker-modified αSyn compared to untreated protein. Moreover, the Coomassie staining in Fig. 2d shows that the DSP/βME method quantitatively retains αSyn on the membrane.

### *In vitro* incubation of lysates with 2 mM DSP followed by βME reduction allows optimal αSyn detection

**Figure 3:**
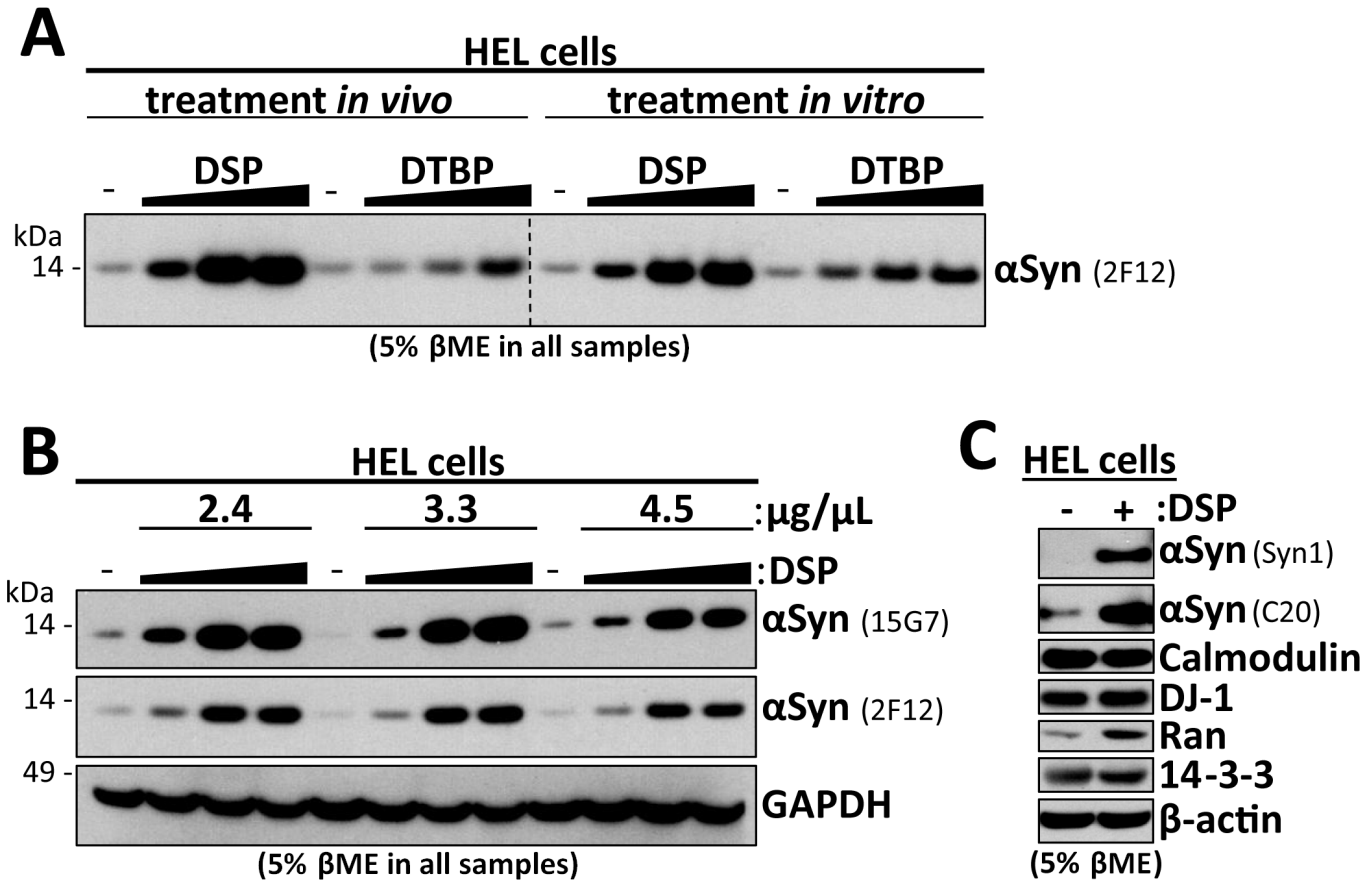
*In vitro* incubation of lysates with 2 mM DSP followed by βME reduction allows optimal αSyn detection. **A.** HEL intact cells (*in vivo*) or lysates (*in vitro*) were incubated with DMSO only (-) as well as gradients of 0.5, 2.0 and 8.0 mM DSP and DTBP, respectively. Cytosols (post-100,000 *g*) were boiled in sample buffer/5% βME and blotted with αSyn antibody 2F12. Identical exposures of the same blot are shown; film was cut at dotted line. **B.** HEL cell cytosols (post-100,000 *g*) at three different protein concentrations (2.4, 3.3., 4.5 µg/µL) were incubated with DMSO only (-) as well as a gradient of 0.5, 2.0 and 8.0 mM DSP and DSG. Samples were normalized to 2.4 µg after quenching, then boiled in sample buffer plus 5% βME and blotted with αSyn antibodies Syn1 and 15G7 as well as an antibody to GAPDH. **C.** HEL cell cytosols (post-100,000 *g*) were treated with DMSO-only (-) or DSP, quenched, boiled in sample buffer plus 5% βME and analyzed by blotting for αSyn (mAb Syn1 and pAb C20), Calmodulin, DJ-1, Ran, 14-3-3 and β-actin.

To optimize the DSP/βME method, we focused on human erythroid leukemia (HEL) cells, which are rich in endogenous αSyn, more consistent, and more readily available than primary neurons or brain samples [7]. We compared the cleavable crosslinkers DSP (spacer length 12.0 Å) and dithiobis[propionimidate] (DTBP, spacer length 11.9 Å) (Fig. 3a). We tested concentration gradients of 0.5, 2.0 and 8.0 mM of each crosslinker vs. DMSO alone (-) and both *in vivo* and *in vitro*. DTBP/βME treatment had a similar but weaker effect as DSP/βME, and DSP concentrations higher than 2 mM had no additional benefit, suggesting saturation of the mechanism. Overall, we observed no major differences between the *in vivo* and *in vitro* treatments (Fig. 3A). As treatment of lysates is generally easier than that of intact cells, we focused on the *in vitro* approach for further method optimization and on the more efficient compound, DSP. We checked whether the protein concentration during *in vitro* crosslinking affected the degree of αSyn detection on Western blots (Fig. 3b) and saw no clear trend with lysate protein concentrations of 2.4, 3.3 and 4.5 µg/µL for the two αSyn antibodies we tested, 2F12 and 15G7, using a DSP gradient paradigm analogous to Fig. 3a. Independent of the lysate protein concentration, we found a strong enhancement of αSyn immunodetection by DSP/βME treatment that plateaued at 2 mM DSP, while immunodetection of GAPDH was unaffected. However, we subsequently chose a cytosol protein concentration range of between 2 and 5 µg/mL for *in vitro* treatments. We next confirmed the strong αSyn enhancement by DSP/βME using two more αSyn antibodies, C20 and Syn1, while the signals of several control proteins were more weakly enhanced (DJ-1, Ran) or unaffected (Calmodulin, 14-3-3, β-actin) (Fig. 3c). All samples shown in Fig. 3 were treated with βME at 5% final concentration, ruling out the possibility that the reducing agent alone is responsible for the increased immunodetection. This was confirmed when we boiled untreated lysates in the presence or absence of βME and subsequently blotted for αSyn (not shown).

### DSP/βME treatment allows facile detection of αSyn from cultured cells

**Figure 4:**
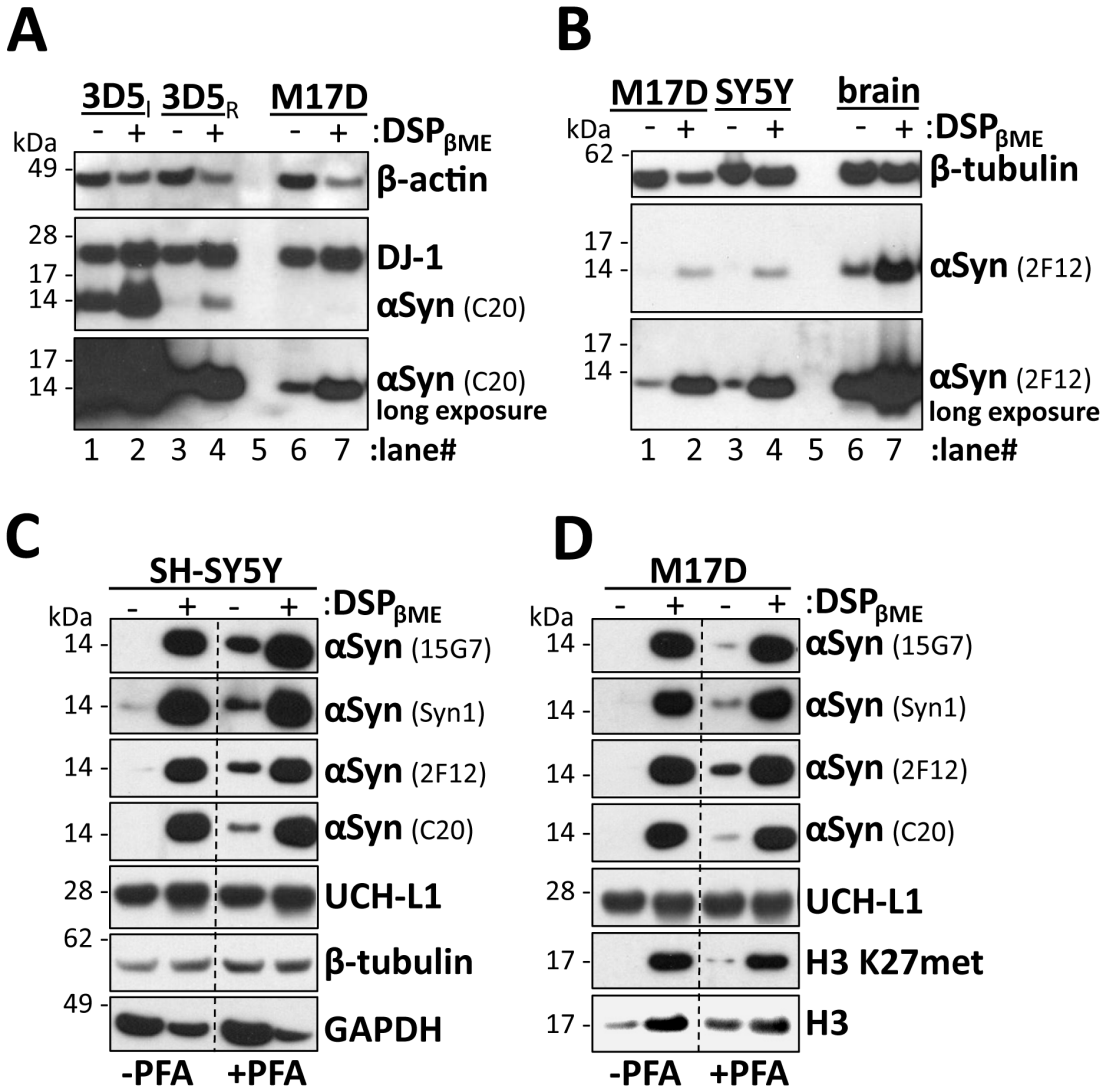
DSP/βME treatment facilitates easy detection of αSyn from cultured cells. **A.** Cytosols (post-100,000 *g*) of 3D5 αSyn tet-off cells in induced (3D5_I_) or repressed state (3D5_R_) as well as lysates of the parental neuroblastoma cell line M17D were crosslinked with 2 mM DSP, followed by boiling in sample buffer with 5% βME. Western blot analysis was performed for αSyn (C20), DJ-1 and β-actin. (Note that the DJ-1 blot shows residual C20 signal from a previous exposure.) **B.** Cytosols (post-100,000 *g*) of neuroblastoma cell lines M17D and SH-SY5Y as well as the PBS-soluble fraction of human brain homogenates were incubated in 2 mM DSP, followed by boiling in sample buffer with 5% βME and blotting for αSyn (2F12) as well as β-tubulin. A long and a short exposure of the 2F12 blot are shown. **C.** Cytosols (post-100,000 *g*) of SH-SY5Y cells were crosslinked with 2 mM DSP, followed by boiling in sample buffer/5% βME and blotting for the indicated antibodies. Membranes were cut at dotted lines after protein transfer and the left half was incubated in 0.4% PFA for 30 min, while the right half was blocked immediately. Membranes were reassembled before film development; PFA-treated and -untreated halves shown were exposed identically. **D.** M17D cells analyzed analogously to SH-SY5Y cells in Fig. 4c. In addition to αSyn and UCH-L1, histone protein H3 was detected using a total H3 as well as an H3 lysine 27-methylation-specific antibody (H3 K27met).

Finally, we tested how well our method detects αSyn in readily available sources whose αSyn expression levels have previously been underestimated, such as cultured cell lines of neuronal origin. We compared the levels of αSyn immunoblot detection with vs. without DSP/βME treatment in the tet-off αSyn overexpressing line 3D5 [14], in the absence (Fig. 4a, top two panels, lanes 1 and 2) and presence (lanes 3 and 4) of doxycycline, to their parental cell line M17D expressing only endogenous αSyn (lanes 6 and 7). Western blotting confirmed very strong expression in induced 3D5 cells compared to doxycycline-repressed 3D5 cells, which still expressed more αSyn than the parental M17D line due to incomplete repression. We observed the αSyn signal-enhancing effect of DSP/βME treatment independently of expression level (compare ‘-‘ lanes to ‘+’ lanes). Detection of the control protein DJ-1 also benefited from DSP/βME treatment to some degree in this experiment, while β-actin detection was again reduced somewhat. Consistent with the report of Lee and Kamitani, PFA treatment alone of the PVDF membranes after transfer already improved detection of endogenous αSyn from these neuroblastoma cells. Nevertheless, additional incubation of the lysates with DSP/βME further enhanced the αSyn signal markedly. We obtained similar results for another commonly studied neuroblastoma cell line, SH-SY5Y (Fig. 4b, lanes 3 and 4). The relative levels of αSyn detected in M17D and SH-SY5Y cells and in human brain homogenates was not changed by DSP/βME treatments, i.e., the αSyn signals were proportionally enhanced regardless of αSyn abundance in the respective source (Fig. 4b, compare long blot exposure for neuroblastoma cells to short exposure for human brains).

To compare systematically the effects of DSP/βME treatment of protein lysates and PFA treatment of blot membranes, we analyzed DSP/βME- and vehicle-treated lysates of SH-SY5Y cells (Fig. 4c) and M17D cells (Fig. 4d) on PVDF membranes with and without PFA treatment. All four αSyn antibodies used routinely in our lab (2F12, 15G7, Syn1, C20) showed the αSyn-enhancing effect of PFA in both cell lines (Figs. 4c and 4d, compare lane 1 to lane 3 in all αSyn panels). DSP/βME treatment, however, led to substantial additional αSyn signal on PFA-treated membranes (Figs. 4c and 4d, compare lane 3 to lane 4 in all αSyn panels). In fact, the strong αSyn signals caused by DSP/βME treatment of the lysates were not significantly enhanced further by additional PFA treatment of the blots (Figs. 4c and 4d, compare lane 2 to lane 4 in all αSyn panels), suggesting that the two treatments do not have additive effects. Furthermore, we tested these same samples on multiple types of transfer membrane – 0.2 µm pore PVDF (our standard), 0.45 µm pore PVDF and 0.2 µm pore nitrocellulose – and found no differences among these membrane materials (data not shown). While signals for most control proteins were either unchanged (UCH-L1, β-tubulin) or reduced (GAPDH) by DSP/βME treatment, we did observe an immunodetection enhancing effect on the histone protein H3 using an antibody to total H3 as well as one specific for trimethyl H3 (lysine 27) (Fig. 4d). This finding is of interest because histone proteins share certain features with αSyn – small size (αSyn: 140 aminoacids; H3: 136 aminoacids) and high lysine content (αSyn: 15; H3: 13) – suggesting that enhancement of immunodetection by lysine modification may be a common theme among small, lysine-rich proteins.

## Discussion

Here, we present a simple method to markedly increase Western blot signals of αSyn by improving its retention on blot membranes. Our method involves protein modification by the cleavable amine-reactive crosslinker DSP followed by reductive cleavage of the crosslinks. In our optimized protocol, intact cells or cell lysates (2–5 µg/µL protein concentration) are treated with 2 mM DSP at 37°C for 30 min, followed by addition of 50 mM Tris (pH 7.6) to quench remaining crosslinker, and then boiling in dodecyl sulfate sample buffer containing 5% βME. Higher concentrations of DSP did not further enhance αSyn immunoreactivity. Beneficial effects of βME treatment alone were ruled out. A recently published method of improving αSyn immunodetection, i.e., incubation of blot membranes in 0.4% PFA [12], is less effective than our DSP-based method, and treatment with PFA in addition to DSP did not improve immunodetection beyond the effect of DSP/βME alone.

The study describing the PFA method begins with the observation that endogenous αSyn in neuroblastoma cells is easily detected by fluorescent immunocytochemistry but not by standard Western blotting, despite the generally higher sensitivity of the latter technique [12]. Noting a previously reported method using the fixative PFA to improve immunodetection of hemoglobin, the authors applied this to Western blots of αSyn and found that the discrepancy between immunocytochemistry and WB was resolved [13]. Regarding the mechanism of the effect, they showed that the PFA method reduced αSyn wash-off from blot membranes. In our recent αSyn study [7], we observed discrepancies between αSyn immunodetection in crosslinked and non-crosslinked samples: αSyn immunodetection was greatly reduced in non-crosslinked samples [7]. With the PFA method, we could partially overcome this problem, suggesting that PFA could partially substitute for crosslinker modification and that the crosslinker, similarly to PFA, improved retention of αSyn on Western blots. However, even with PFA treatment of blot membranes, we found that untreated or lightly crosslinked samples routinely yielded lower αSyn monomer immunodetection than more highly crosslinked samples (see Fig. 1). This indicated that crosslinker modification improved retention of αSyn to a greater extent than the PFA method, even when the crosslinker is cleaved to yield just monomers. Indeed, DSP/βME treatment generally led to stronger αSyn signals relative to PFA treatment alone, while the PFA method was always superior to completely untreated samples (e.g., Fig. 4c and 4d). Nearly complete retention on membranes of recombinant αSyn treated with DSP/βME after hours of washing, in contrast to the substantial loss of untreated protein, confirms that the DSP/βME method, like PFA treatment, effects stronger immunodetection by enhancing retention on the blot membrane rather than through epitope accessibility. Moreover, the close to complete retention observed suggests that further enhancement of αSyn immunodetection may not be necessary.

The mechanism underlying the reported PFA effect is not self-evident [13]. The chemical inertness of PVDF and nitrocellulose makes formation of covalent bonds with αSyn, by reaction with PFA, improbable. As proteins bind to blot membranes through non-covalent, primarily hydrophobic interactions [21], [22], small hydrophilic proteins like αSyn are not expected to bind as strongly as larger, more hydrophobic proteins. It is thus possible that PFA exerts its stabilizing effect on αSyn through formation of covalent bonds with other proteins that adhere more firmly to the blot membrane. However, Lee and Kamitani showed that the PFA method is effective even with purified αSyn. We similarly show that purified αSyn is retained on membranes to a greater extent with DSP/βME treatment than without, indicating that modification of lysine residues is sufficient for retention (Fig. 2d). Another amine-reactive, reducible crosslinker, DTBP, also enhanced αSyn immunoblotting though not as greatly as DSP; this difference may be due to the positive charge of the amidine product of the DTBP-lysine reaction instead of the uncharged amide product of the DSP-lysine reaction. Furthermore, the strong effect of DSP/βME treatment, despite cleavage of the crosslinker, makes intermolecular crosslinking of αSyn an unlikely explanation for the PFA effect.

We hypothesize that both PFA and DSP/βME effects occur through a combination of: a) masking the positive charges of lysines (of which αSyn has 15), thereby increasing αSyn hydrophobicity and blot membrane binding; and b) adding to αSyn a net hydrophobic moiety large enough to confer stronger interaction with the blot membrane. As non-immunological stains - Coomassie Blue dye - also show that crosslinker-modified αSyn can be fully retained on membranes, unlike unmodified αSyn (Fig. 2d), the enhanced immunodetection is probably not mediated by improved epitope accessibility. The closely similar results with all αSyn (as well as β- and γSyn) antibodies we tested further corroborate this conclusion. It was surprising that, with some exceptions such as GAPDH, modification of lysines did not negatively interfere with immunodetection of most proteins tested. The DSP/βME effect was even observed for the Syn1 antibody, whose recognized epitope contains lysines. Based on our experience with αSyn, we postulated that other small, lysine-rich proteins like the αSyn homologs βSyn and γSyn and histone-family proteins might similarly benefit from DSP/βME treatment. Indeed, we observed enhanced Western blot detection upon DSP/βME treatment for all three synucleins and the histone H3 (other histones not tested) (Figs. 2b and 4d).

Our results support a conclusion from the Lee and Kamitani paper, that the field must reconsider the notion that immortalized cell lines are necessarily very low in αSyn expression. Our data show that they indeed have lower expression levels than brain homogenates, but αSyn is believed to constitute up to 1% of cytosolic proteins in neural tissue [23], and thus, the relatively lower levels detected by our method for the neuroblastoma cell lines M17D and SH-SY5Y may still be relatively high compared to other cellular proteins. Therefore, we propose using our new method in future studies of αSyn using Western blotting, at least in comparison to traditional Western blot protocols or the PFA-alone method, especially when analyzing sources with low levels or rare forms of αSyn such as secreted αSyn, low abundance splice variants, or possibly rare modifications of αSyn. Similarly, our method ensures detection of nearly all αSyn present at 14 kDa after transfer, allowing more quantitatively correct study of, for example, the relative abundance of high molecular weight aggregates and unaggregated oligomers by Western blotting (as shown and discussed for the PFA method in [12]). Finally, we propose DSP/βME treated samples to be the correct control to estimate total αSyn levels in comparison to the different oligomeric species trapped by uncleaved crosslinkers when performing crosslinking analyses of physiological αSyn oligomerization. If one used a vehicle-only control instead of DSP/βME, the consequent loss of αSyn monomer would give the impression that the crosslinked sample contained additional αSyn that was generated by the crosslinking. It should be remembered, however, that treatment with DSP/βME or PFA may mask antibody epitopes, although we did not observe such an effect with any of our αSyn, βSyn, γSyn or histone antibodies, nor for most of our control antibodies. However, each antibody needs to be assessed independently, regardless of its target. An additional caveat of our method is that certain standard biochemical analyses of protein lysates treated with DSP/βME may be altered, such as assays to determine protein concentrations or Ponceau membrane staining, in which masking of lysines interferes with binding of the dye to proteins. To overcome these limitations, we suggest normalizing samples before DSP treatment and visualizing proteins on blot membranes by methods less dependent on the presence of positive charges.

In conclusion, the present method should be useful for all synuclein researchers who want to study, by Western blotting, the total synuclein in a given cellular, tissue or *in vitro* sample rather than just the small fraction detected using standard techniques.
